# Impact of Navier’s slip and chemical reaction on the hydromagnetic hybrid nanofluid flow and mass transfer due to porous stretching sheet

**DOI:** 10.1038/s41598-022-14692-y

**Published:** 2022-06-21

**Authors:** U. S. Mahabaleshwar, T. Anusha, O. Anwar Bég, Dhananjay Yadav, Thongchai Botmart

**Affiliations:** 1 Department of Mathematics, Davangere University, Shivagangothri, Davangere, 577 007 India; 2 School of Science, Engineering and Environment, Aeronautical and Mechanical Engineering, University of Salford, Manchester, UK; 3 Department of Mathematical and Physical Sciences, University of Nizwa, Nizwa, Oman; 4 Department of Mathematics, Faculty of Science, Khon Kaen University, Khon Kaen, 40002 Thailand

**Keywords:** Engineering, Mathematics and computing, Physics

## Abstract

Hybrid nanofluids (HNFs) comprise combinations of different nanoparticles suspended in base fluid. Applications of such nanofluids are rising in the areas of energy and biomedical engineering including smart (functional) coatings. Motivated by these developments, the present article examines theoretically the magnetohydrodynamic coating boundary layer flow of HNFs from a stretching sheet under the transverse magnetic field in porous media with chemically reactive nanoparticles. Darcy’s law is deployed. Momentum slips of both first and second order are included as is solutal slip. The transformed boundary value problem is solved analytically. Closed form solutions for velocity are derived in terms of exponential functions and for the concentration field in terms of incomplete Gamma functions by the application of the Laplace transformation technique. The influence of selected parameters e.g. suction/injection, magnetic field and slips on velocity and concentration distributions are visualized graphically. Concentration magnitudes are elevated with stronger magnetic field whereas they are suppressed with greater wall solutal slip. Magnetic field suppresses velocity and increases the thickness of the hydrodynamic boundary layer. The flow is accelerated with reduction in inverse Darcy number and stronger suction direct to reduce in skin friction. The concentration magnitudes are boosted with magnetic field whereas they are depleted with increasing solutal slip. The analysis provides a good foundation for further investigations using numerical methods.

## Introduction

In recent years NF^1^ have mobilized significant interest owing to their superior performance in many technologies including bio-microfluidics, aerospace, environmental and energy systems^2–8^. NF are colloidal suspensions engineered at the nanoscale and comprise conventional base fluids e. g. water, doped with metallic (e. g. zinc, titanium, iron, copper, gold, silver and their oxides) or non-metallic (carbon based e. g. silicates, graphene, diamond etc.) nanoparticles (spheres, rods, tubes, shells, ellipsoids etc.). *Magnetic nanofluids*^9^ are another subset of modern NF which feature electrically conducting nanoparticles and invoke magnetohydrodynamic (MHD)^10^ effects, allowing thermal/mass transport to be manipulated via external magnetic fields. These have been examined in many applications and recent works in this regard include Bég et al*.*^11^ (smart orthopaedic magnetic NF films) and Li et al*.*^12^ (magnet core/shell L1_0_-CoPt/Pt-water NF in Proton exchange membrane fuel cells).

Functional nanomaterials, of which magnetic NF are an example, offer improved corrosion and abrasion resistance in the new generation of “smart nano-coatings”^13^. The manufacturing of such NF smart coatings frequently involves the stretching of a sheet over a metallic substrate or enrobing. This area offers many interesting features which can be studied with fluid mechanics and applied mathematical methods and has therefore stimulated considerable interest in recent years. Mahabaleshwar et al*.*^14^ investigated the hydromagnetic NF flow with mass flux (suction) observing that an increment in Chandrasekhar number (square of the Hartmann number) strongly decelerates the flow and furthermore that a substantial modification in flow is induced with different nanoparticle types. Aneja et al*.*^15^ adopted a variational finite element method and Buongiorno’s 2-component nanoscale model to compute the impact of non-uniform magnetic field on aqueous NF stretching sheet flow containing motile gyrotactic micro-organisms from a tilted surface with Ohmic dissipation (Joule heating) as a model of smart solar coatings. They observed that strong retardation in the flow is produced with greater inclination and magnetic field strength whereas temperatures, motile micro-organism density number (bioconvection species concentration) and nanoparticle concentration are boosted. Hamad^16^ derived closed-form solutions for MHD NF boundary layer flow from a semi-infinite vertical stretching surface under static transverse magnetic field, studying in detail the impact of nanoparticle solid volume fraction and magnetic field on Nusselt number and skin friction coefficient. Hsiao^17^ used the Keller box finite difference scheme to simulate the hydromagnetic boundary layer flow in micropolar NF flow from a stretching sheet. He found that stronger magnetic parameter depresses velocity magnitudes and Nusselt number whereas reverse effects in shear stress, wall couple stress (micro-rotation gradient), temperature and nanoparticle concentration.

The above studies were generally confined to unitary magnetic NF, in which a single magnetic nanoparticle is deployed in the base fluid. However, engineers have also explored combinations of different nanoparticles in base fluids, and these are known as *hybrid nanofluids (HNFs)*. It has been observed from both theoretical and experimental studies^18–20^ that HNF achieve even better thermal and mass diffusion enhancement characteristics than conventional (unitary i.e. single nanoparticle material) NF. Applications of HNF have been explored in biocompatible nano-hemodynamics, mechanical heat sinks, helical and plate heat exchangers and also smart thin film coatings. Examples of HNF studied include TiO_2_–CuO/Ethylene glycol nanofluid^21^ and aqueous silica–alumina HNF^22^. Lund et al.^23^ showed that the heat transfer rate and mass transfer in HNFs is higher than normal NFs. Hayat and Nadeem^24^ examined 3-D rotating flow of an aqueous Ag–CuO HNF, also noting superior performance of the hybrid mixture compared with unitary NF. Other studies have considered combinations of graphite and zinc diamond nanoparticles in hybrid designs. Prakash et al.^25^ investigated electro-osmotic propulsion of HNF (titania, alumina or copper metallic nanoparticles in water) in a deformable conduit using All these studies confirm that improved thermo-mass diffusion performance is attained with HNF. HNF transport from stretching sheets of relevance to materials processing systems has also been examined by a variety of researchers. Aly and Pop^26^ computed the MHD flow of aqueous HNF based on shrinking/stretching sheets, considering dual solutions. Khan et al.^27^ studied HNF TiO_2_–Cu/H_2_O stretching sheet flow, observing superior performance to unitary Cu-Water NF.

In many coating applications, a porous medium can be deployed to achieve improved flow control during deposition processes^28^. The implementation of porous media is useful in stretching flows where, as explained earlier, the nanocoating is extended over a substrate (e.g. metallic component). Porous media also feature extensively in biomedical applications. Some interesting studies of magnetic NF flows in porous media include Manh et al*.*^29^ deployed a CVFEM to simulate hydromagnetic nanoparticle (Fe_3_O_4_ + MWCNT) aqueous NF hydromagnetic flow in a non-Darcy porous media with radiative flux and buoyancy effects. They noted that average Nusselt number is boosted markedly with thermal buoyancy effect (Rayleigh number) and permeability effect (Darcy number) whereas it is decreased with magnetic Hartmann number and furthermore velocity field is induced with higher Hartmann number.

While most NF studies have considered thermal aspects, very few have considered solely the momentum and mass diffusion in nano-coating boundary layer flow. In mass transfer coating operations, chemical reactions frequently occur, and these can be manipulated to produce specific characteristics in nano-coatings which are critical for their subsequent selection in different applications. This is particularly of relevance to the sol–gel method of nanocoating synthesis wherein colloidal nanoparticles are generated from the liquid phase. This chemical method is based on hydrolysis or condensation reactions and it has been shown^30^ that with carefully organized reactants, nanosized particles precipitate and produce coatings which exhibit exceptional advantages such as versatility and easy shaping. Chemical reactions may be constructive or destructive. They may also be homogeneous or heterogeneous. Mathematical models of reactive coating flows have got some interest in recent days. Uddin et al*.*^31^ used Lie group algebraic methods and MAPLE quadrature to analyze the 2-D hydromagnetic viscous flow. They obtained that velocity and temperature is enhanced with rising order of chemical reaction whereas nanoparticle volume fraction (concentration) is condensed.

In numerous materials fabrication systems slip effects are known to arise at the wall (e.g. conveyor belt). Slip is generally associated with molecular dynamics in the fluid near the boundary and leads to non-adherence of coatings to substrates. These effects can significantly alter heat, mass and momentum characteristics in coating extrusion. The boundary value problems associated with such flows have stimulated some interest in recent years and in addition to momentum slip, thermal and mass jump (slip) effects are also addressed. Bég et al.^32^ used the PSPICE network electro thermal code to compute the slip effects on Von Karman swirling hydromagnetic convective flow from a rotating disk with wall transpiration and radiative heat transfer. Mahabaleshwar et al*.*^33^ investigates the stretching sheet convective-radiative flow of a short memory viscoelastic fluid with Navier wall slip in Darcian porous media. Mahabaleshwar et al.^34^ modeled the problem of axisymmetric flow over stretching surface with slip; since this boundary value problem did not yield analytical solutions it was therefore solved with a DTM in combination with Padé approximations to accelerate convergence. NF slip boundary layer flows have been investigated by Hakeem et al*.*^35^ who considered second order slip in radiative magnetic NF flow from both extending and contracting sheets. Shukla et al*.*^36^ obtained homotopy solutions for entropy generation in time-dependent stagnation slip flow of a reactive NF with both electrical and magnetic fields. Further studies include Govindaraju et al.^37^ (for silver-water NFs), Bhatti*et al.*^38^ (for magnetic Fe_3_O_4_-water-based NF with quadratic sheet stretching and cross diffusion effects), Babu and Sandeep^39^ (for water-graphene NF judge against with water-magnetite NF), Uddin et al*.*^40^ (for MHD bioconvection NF coating flows), Tulu and Ibrahim^41^ (for CNT-ethylene glycol NF swirling disk flow) and Shukla et al*.*^42^ (for entropy generation in magnetic silver/zinc NF external coating boundary layers on a vertical cylinder). Recently in 2022 Mahabaleshwar et al.^43^ examined the non-Newtonian fluid flow over porous sheet by considering the effect of magnetic field and heat transfer with heat source/sink. Further found the different solution methods. Mahabaleshwar et al.^44^ examined the steady flow with HNF with mass suction and found the solution in algebraically decaying form. Recently Anusha et al.^45^ respectively investigates the MHD flow and mass transfer due to porous medium with hybrid nanoparticles. Mahabaleshwar et al.^46^ made the article on the MHD flow with CNTs and investigated the effect of mass transpiration and radiation. Shanmugapriya et al.^47^ investigate the enhancement of heat and mass transfer due to HNF SWCNTs and MWCNTs in water. Andersson^48^ studied the mass transfer of chemical reactive species due to stretching sheet. Singh et al.^49^ examine the nonlinear MHD flow.

Although in many boundary value problems, numerical methods are frequently utilized, it is also possible to use the method of LT to derive analytical solutions for NF coating boundary layer flows. Saleh et al.^50^ investigated convection in CNT NF with cancer tumor treatment applications using LT. Ebaid and Sharif^51^ studied thermo-magnetic CNT NF flow with LT methods. Bhullar et al*.*^52^ has provided a detailed appraisal of applications and properties of LT methods in fluid dynamics and other areas of engineering sciences. These works on finding analytical solution by using LT motivated to carry out the present research. And there are many applications of the present work in many areas such as, medical field for treatment of cancer tumor wherein injecting nanoparticles into the blood and magnetic field is applied as an external force. The flow along the artery is the boundary layer flow. And many applications in heating and cooling systems, solar energy, space, processing plants, MHD power generators, spinning of filaments, geothermal recovery, electrochemical process, catalytic reactors etc.

In the current article, a mathematical model is introduced for the convective momentum and mass transfer in MHD HNF flow from a stretching sheet adjacent to a porous medium with chemical reaction. Navier’s slip conditions in addition to thermal and mass (nanoparticle) wall slip are also included. Closed form solutions for velocity are derived in terms of exponential functions and for the concentration field in terms of incomplete Gamma functions by the application of the LT technique. The influence of selected parameters e.g. suction/injection, magnetic field and slip parameters on velocity and concentration distributions are visualized graphically. The computations are relevant to smart functional magnetic nanofluid coating applications.

## Mathematical model

**Figure 1:**
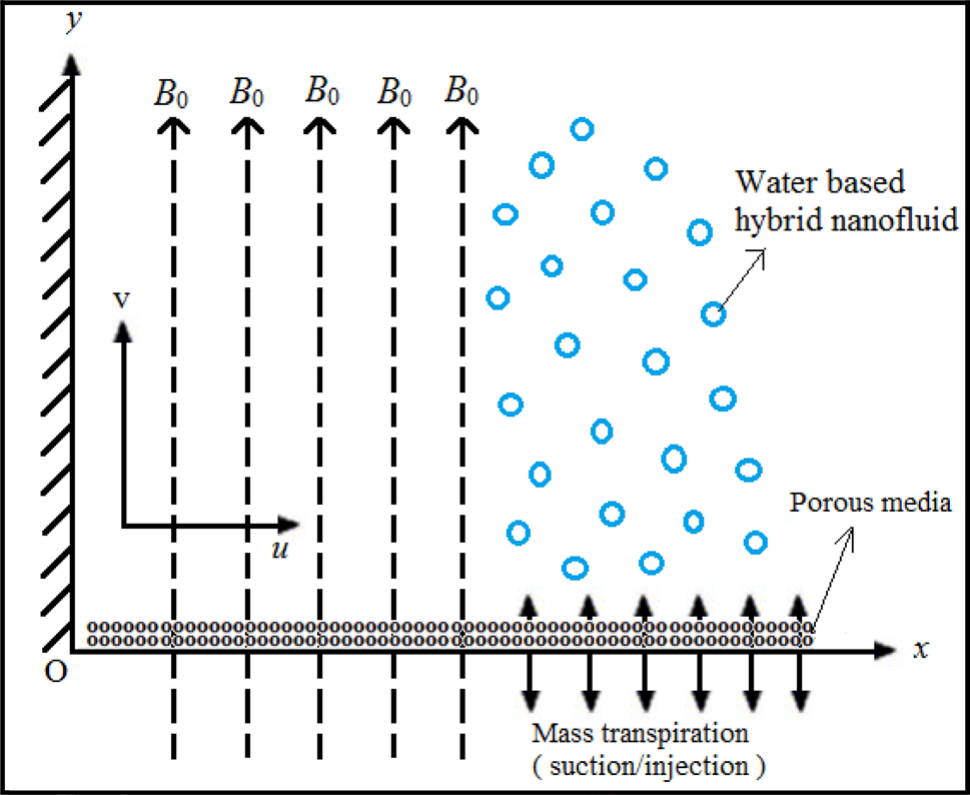
Schematic of reactive MHD nanofluid coating boundary layer flow in porous media.

Consider the incompressible Newtonian HNF flow due to a linearly stretched semi-infinite sheet (coating) embedded in porous media with mass transpiration and chemical reaction. Navier’s momentum slip^53^ and also nanoparticle concentration (solutal) slip are included. The sheet is stretched in the *x*-direction and the *y*-axis is perpendicular to the plane of stretching. A uniform magnetic field with strength *B*_0_ is applied in the *y*-direction. Magnetic induction effects are negated (magnetic Reynolds number is sufficiently small such that the magnetic field is not distorted). The porous medium is non-deformable, assumed to be *isotropic* (constant permeability in all directions) and homogenous and Darcy’s law is assumed. The NF is also assumed to be dilute and a first order homogenous destructive chemical reaction is considered. Under these approximations and the boundary-layer assumption, the model of the present flow is taken as the following system of coupled, nonlinear PDE’s (see Saleh et al.^50^) (Fig. 1):

1 $$ u_{x} + {\text{v}}_{y} = 0, $$

2 $$ uu_{x} + {\text{v}}u_{y} = \nu_{eff} u_{yy} - \frac{{\sigma_{hnf} B_{0}^{2} }}{{\rho_{hnf} }}u - \frac{{\mu_{hnf} }}{{\kappa \rho_{hnf} }}u, $$

3 $$ uC_{x} + {\text{v}}C_{y} = D_{B} C_{yy} - k_{c} \left( {C - C_{\infty } } \right), $$

The associated B.C.s are:

4a $$ u\left( {x,0} \right) = ax + u_{slip} ,\,\,{\text{v}}\left( {x,0} \right) = {\text{v}}_{w} ,\,\,u\left( {x,\infty } \right) \to 0, $$

4b $$ C\left( {x,0} \right) = C_{w} + K_{1} C_{y} \left( {x,0} \right),\,\,C\left( {x,\infty } \right) \to C_{\infty } , $$

In Eq. (4a), $u_{slip}$ is the wall slip velocity which is taken as (see Wu^54^):

5 $$ u_{slip} = \frac{2}{3}\left[ {\frac{{3 - \gamma m^{3} }}{\gamma } - \frac{{3\left( {1 - m^{2} } \right)}}{2K}} \right]\alpha u_{y} - \frac{1}{4}\left[ {m^{4} + \frac{2}{{K^{2} }}\left( {1 - m^{2} } \right)} \right]\alpha^{2} u_{yy} , $$

This may be re-written as follows:

6 $$ u_{slip} = L_{1} u_{y} + L_{2} u_{yy} , $$

Here, $0 \le \gamma \le 1$ is the coefficient of momentum accommodation, further the molecular free path $\alpha$ is positive, which implies that $L_{1} \ge 0$ and $L_{2} \le 0$. *m* is the minimum value among the numbers 1/*K* and 1 which clearly implies $0 \le m \le 1$ for any *K*, where *K* is the Knudsen number which is valid for arbitrary values as elaborated in Wu^54^. Additionally, the applications of Knudsen number can be found in^54^. The primitive form of the boundary layer equations is challenging to solve. It is therefore pertinent to introduce scaling transformations. Consider suitable similarity transformations with stream function which following Hamad^16^ may be defined as follows:

7 $$ \psi = \sqrt {a\nu_{f} } xf\left( \eta \right)\quad {\text{and}}\quad \eta = \sqrt {\frac{a}{{\nu_{f} }}} y, $$

The velocities along *x*- and *y*-directions respectively emerge in terms of these transformations as:

8 $$ u = axf_{\eta } \left( \eta \right)\,\,\,\,{\text{and}}\,\,\,\,{\text{v}} = - \sqrt {a\nu_{f} } f\left( \eta \right), $$

Further the transformation for concentration is taken as:

9 $$ \phi \left( \eta \right) = \frac{{C - C_{\infty } }}{{C_{w} - C_{\infty } }}, $$

On applying (7) to (9) in the momentum and species diffusion boundary layer Eqs. (2) and (3) we obtain:

10 $$ \delta_{1} \Lambda f_{\eta \eta \eta } + \delta_{1} \left( {ff_{\eta \eta - } f_{\eta }^{2} } \right) - \left( {\delta_{3} M + \delta_{2} Da^{ - 1} } \right)f_{\eta } = 0, $$

11 $$ \phi_{\eta \eta } + Scf\phi_{\eta } = \beta Sc\phi , $$

The B.C.s (4a) and (4b) become:

12a $$ f\left( 0 \right) = V_{C} ,\,\,\,f_{\eta } \left( 0 \right) = 1 + S_{1} f_{\eta \eta } \left( 0 \right) + S_{2} f_{\eta \eta \eta } \left( 0 \right),\,\,\,f_{\eta } \left( \infty \right) = 0, $$

12b $$ \phi \left( 0 \right) = 1 + S_{3} \phi_{\eta } \left( 0 \right),\,\,\,\phi \left( \infty \right) = 0, $$

Here, all physical parameters are defined in the nomenclature and are in accordance with Andersson and Bech^55^, Siddheshwar et al*.*^56^ and Andersson et al*.*^48^.

## Momentum problem

The exact solutions for the momentum Eq. (10) with the relevant B.Cs from (12a,b) can be expressed in the form:

13 $$ f\left( \eta \right) = A + Bexp\left( { - \lambda \eta } \right), $$

On using B.Cs (12a) to determine the constants, the solution will become:

14 $$ f\left( \eta \right) = V_{C} - \frac{{\left[ {1 - exp\left( { - \lambda \eta } \right)} \right]}}{{\lambda \left( {S_{2} \lambda^{2} - S_{1} \lambda - 1} \right)}}, $$

The wall skin friction (dimensionless surface shear stress) is given by:

15 $$ - f_{\eta \eta } \left( 0 \right) = - \frac{\lambda }{{S_{2} \lambda^{2} - S_{1} \lambda - 1}}, $$

Here the unknown $\lambda$ can be determined from the following equation which is obtained by using Eq. (13) in (10):

16 $$ \begin{aligned} & \delta_{1} \Lambda S_{2} \lambda^{4} - \delta_{1} \left( {\Lambda S_{1} + V_{C} S_{2} } \right)\lambda^{3} - \left( {\delta_{1} \Lambda - \delta_{1} V_{C} S_{1} + \delta_{3} MS_{2} + \delta_{2} Da^{ - 1} S_{2} } \right)\lambda^{2} \\ & \quad + \left( {\delta_{1} V_{C} + \delta_{3} MS_{1} + \delta_{2} Da^{ - 1} S_{1} } \right)\lambda + \left( {\delta_{1} + \delta_{3} M + \delta_{2} Da^{ - 1} } \right) = 0 \\ \end{aligned} $$

Next, we implement the method of LT to find the simple solution in terms of incomplete Gamma function.

## Mass transfer problem

Using Eq. (13) in the dimensionless nanoparticle species conservation Eq. (7) the mass transfer Eqn. is rendered as follows:

17 $$ \phi_{\eta \eta } + Sc\left[ {A + Bexp\left( { - \lambda \eta } \right)} \right]\phi_{\eta } = \beta Sc\phi , $$

By taking the new variable $\xi = exp\left( { - \lambda \eta } \right)$, Eq. (17) takes the form:

18 $$ \xi \phi_{\xi \xi } + \left[ {1 - \frac{Sc}{\lambda }\left( {A + B\xi } \right)} \right]\phi_{\xi } = \frac{\beta Sc}{{\lambda^{2} }}\frac{1}{\xi }\phi , $$

The B.C.s (12b) assumes the form:

19 $$ \phi \left( 1 \right) = 1 + S_{3} \phi_{\xi } \left( 1 \right),\,\,\,\phi \left( 0 \right) = 0, $$

Introducing the substitutions:

20 $$ 1 - \frac{ScA}{\lambda } = \chi_{1} ,\quad \frac{ScB}{\lambda } = \chi_{2} ,\quad {\text{and}}\quad \frac{\beta Sc}{{\lambda^{2} }} = \chi_{3} , $$

It follows that Eq. (18) becomes:

21 $$ \xi \phi_{\xi \xi } + \left[ {\chi_{1} - \chi_{2} \xi } \right]\phi_{\xi } = \chi_{3} \frac{1}{\xi }\phi , $$

To solve Eq. (21), we deploy LT to obtain:

22 $$ S\left( {\chi_{2} - S} \right)\phi_{S} \left( S \right) + \left[ {\chi_{2} + S\left( {\chi_{1} - 2} \right)} \right]\phi \left( S \right) = \chi_{3} \int_{S}^{\infty } {\phi \left( S \right)dS} , $$

The solution of Eq. (22) for the non-reactive species i.e. for $\beta = 0$ is:

23 $$ \phi \left( S \right) = \frac{C}{{S\left( {S - \chi_{2} } \right)^{{\left( {1 - \chi_{1} } \right)}} }} $$

In order to apply inverse LT,$\chi_{1} < 1$ and here $\phi \left( S \right) = L\left[ {\phi \left( \xi \right)} \right]$ so that Eq. (23) gives:

24 $$ \phi \left( \xi \right) = \frac{{C\xi^{{ - \chi_{1} }} exp\left( {\chi_{2} \xi } \right)}}{{\Gamma \left[ {1 - \chi_{1} } \right]}}, $$

By applying the convolution property of LT in Eq. (24) gives:

25 $$ \phi \left( \xi \right) = \frac{C}{{\left( { - \chi_{2} } \right)^{{\left( {1 - \chi_{1} } \right)}} }}\frac{{\Gamma \left[ {1 - \chi_{1} ,0, - \chi_{2} \xi } \right]}}{{\Gamma \left[ {1 - \chi_{1} } \right]}}, $$

Using the B.C.s in (19) to find the constant in Eq. (25) yields:

26 $$ \phi \left( \xi \right) = \frac{{\Gamma \left[ {1 - \chi_{1} ,0, - \chi_{2} \xi } \right]}}{{\Gamma \left[ {1 - \chi_{1} ,0, - \chi_{2} } \right] + S_{3} exp\left( {\chi_{2} } \right)\left( { - \chi_{2} } \right)^{{\left( {1 - \chi_{1} } \right)}} }}, $$

In terms of the similarity variable $\eta$ Eq. (26) becomes:

27 $$ \phi \left( \eta \right) = \frac{{\Gamma \left[ {1 - \chi_{1} ,0, - \chi_{2} exp\left( { - \lambda \eta } \right)} \right]}}{{\Gamma \left[ {1 - \chi_{1} ,0, - \chi_{2} } \right] + S_{3} exp\left( {\chi_{2} } \right)\left( { - \chi_{2} } \right)^{{\left( {1 - \chi_{1} } \right)}} }}. $$

This defines the exact analytical solution for the concentration field which in terms of incomplete gamma functions may be expressed as:

28 $$ \phi_{\eta } \left( \eta \right) = \frac{{\lambda \left( { - \chi_{2} exp\left( { - \lambda \eta } \right)} \right)^{{1 - \chi_{1} }} exp\left[ {\chi_{2} exp\left( { - \lambda \eta } \right)} \right]}}{{\Gamma \left[ {1 - \chi_{1} ,0, - \chi_{2} } \right] + S_{3} exp\left( {\chi_{2} } \right)\left( { - \chi_{2} } \right)^{{\left( {1 - \chi_{1} } \right)}} }}, $$

The Sherwood number (dimensionless mass transfer rate at the wall i.e. sheet) takes the form:

29 $$ - \phi_{\eta } \left( 0 \right) = - \frac{{\lambda \left( { - \chi_{2} } \right)^{{1 - \chi_{1} }} exp\left( {\chi_{2} } \right)}}{{\Gamma \left[ {1 - \chi_{1} ,0, - \chi_{2} } \right] + S_{3} exp\left( {\chi_{2} } \right)\left( { - \chi_{2} } \right)^{{\left( {1 - \chi_{1} } \right)}} }}. $$

## Results and discussion

**Figure 2:**
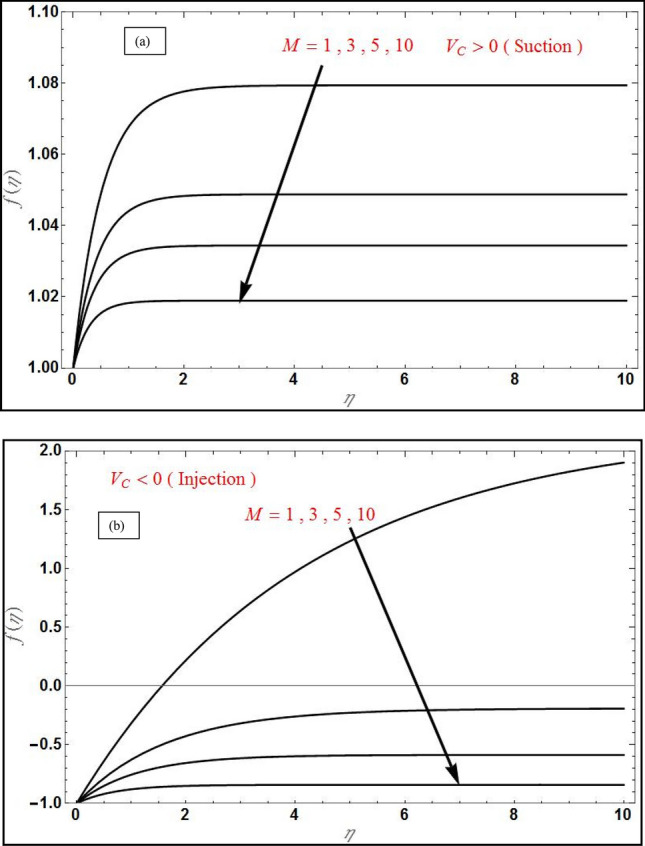
Transverse velocity profile $f\left( \eta \right)$ for varying magnetic parameter $\left( M \right)$ for (**a**) suction $\left( {V_{C} = 1} \right)$ and (**b**) injection $\left( {V_{C} = - 1} \right)$ with $Da^{ - 1} = \Lambda = 1$, $S_{1} = 1,S_{2} = - 1$, $\varphi_{1} = 0.1, \varphi_{2} = 0.04$.

**Figure 3:**
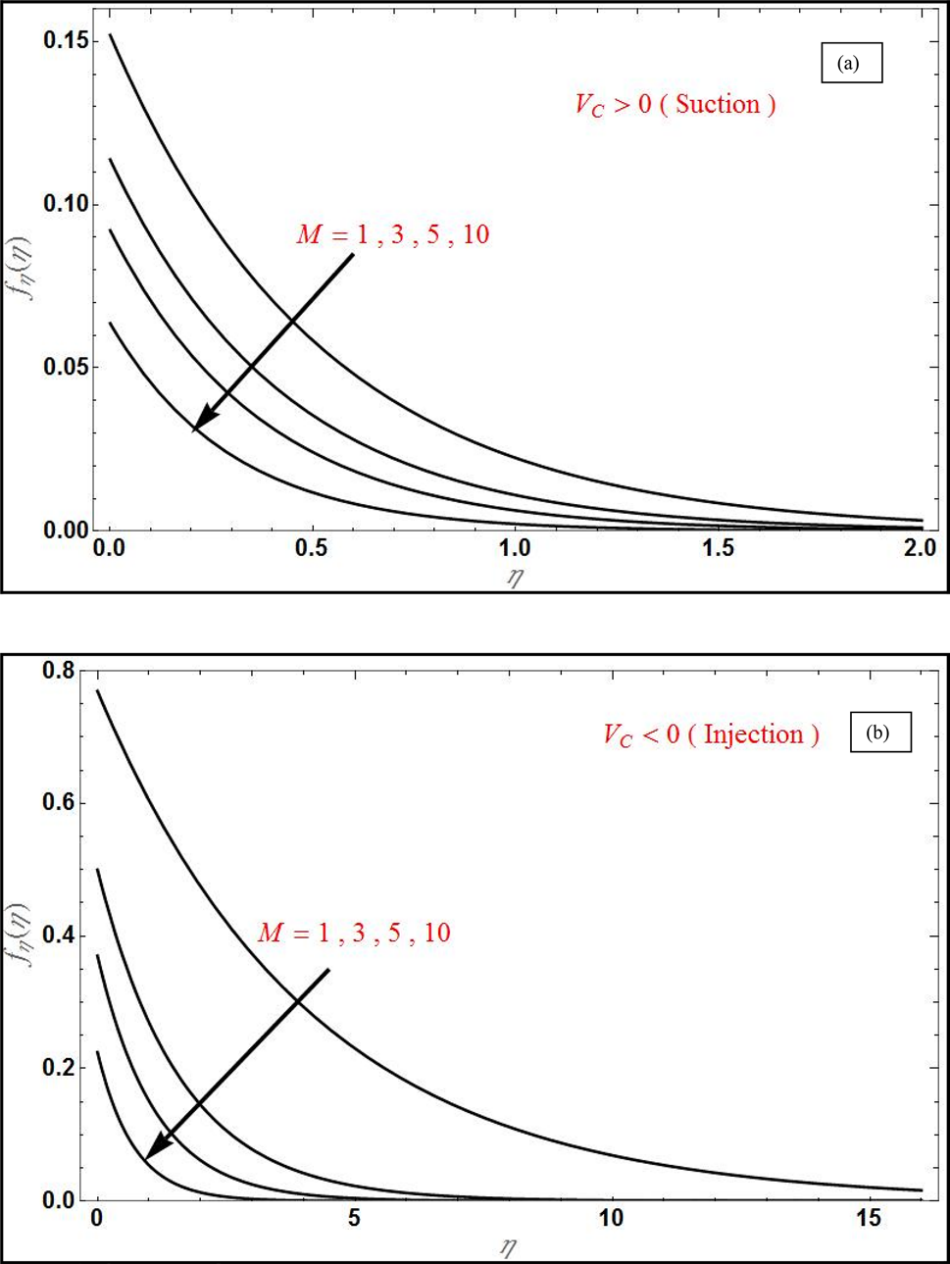
Axial velocity profile $f_{\eta } \left( \eta \right)$ for varying magnetic parameter $\left( M \right)$ for (**a**) suction $\left( {V_{C} = 1} \right)$ and (**b**) injection $\left( {V_{C} = - 1} \right)$ with $Da^{ - 1} = \Lambda = 1$, $S_{1} = 1,S_{2} = - 1$, $\varphi_{1} = 0.1,\varphi_{2} = 0.04$.

**Figure 4:**
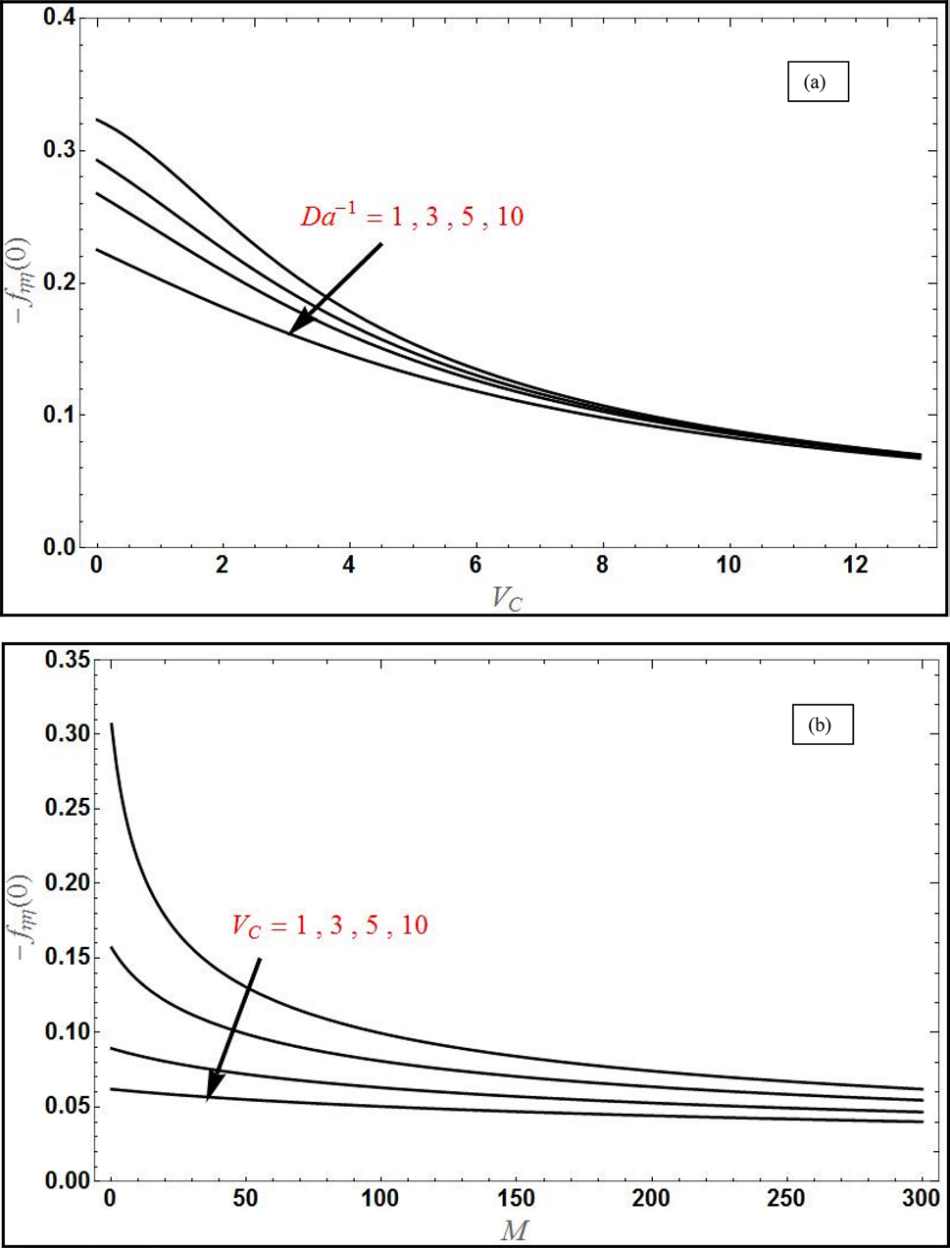
The behavior of skin friction $- f_{\eta \eta } \left( 0 \right)$ in (**a**) as a function of $V_{C}$ for varying value of $\left( {Da^{ - 1} } \right)$ with $M = 1$ and in (**b**) as a function of *M* with different $V_{C}$ with $Da^{ - 1} = 1$ and $\Lambda = 1$, $S_{1} = 1,S_{2} = - 1$, $\varphi_{1} = 0.1,\varphi_{2} = 0.04$.

**Figure 5:**
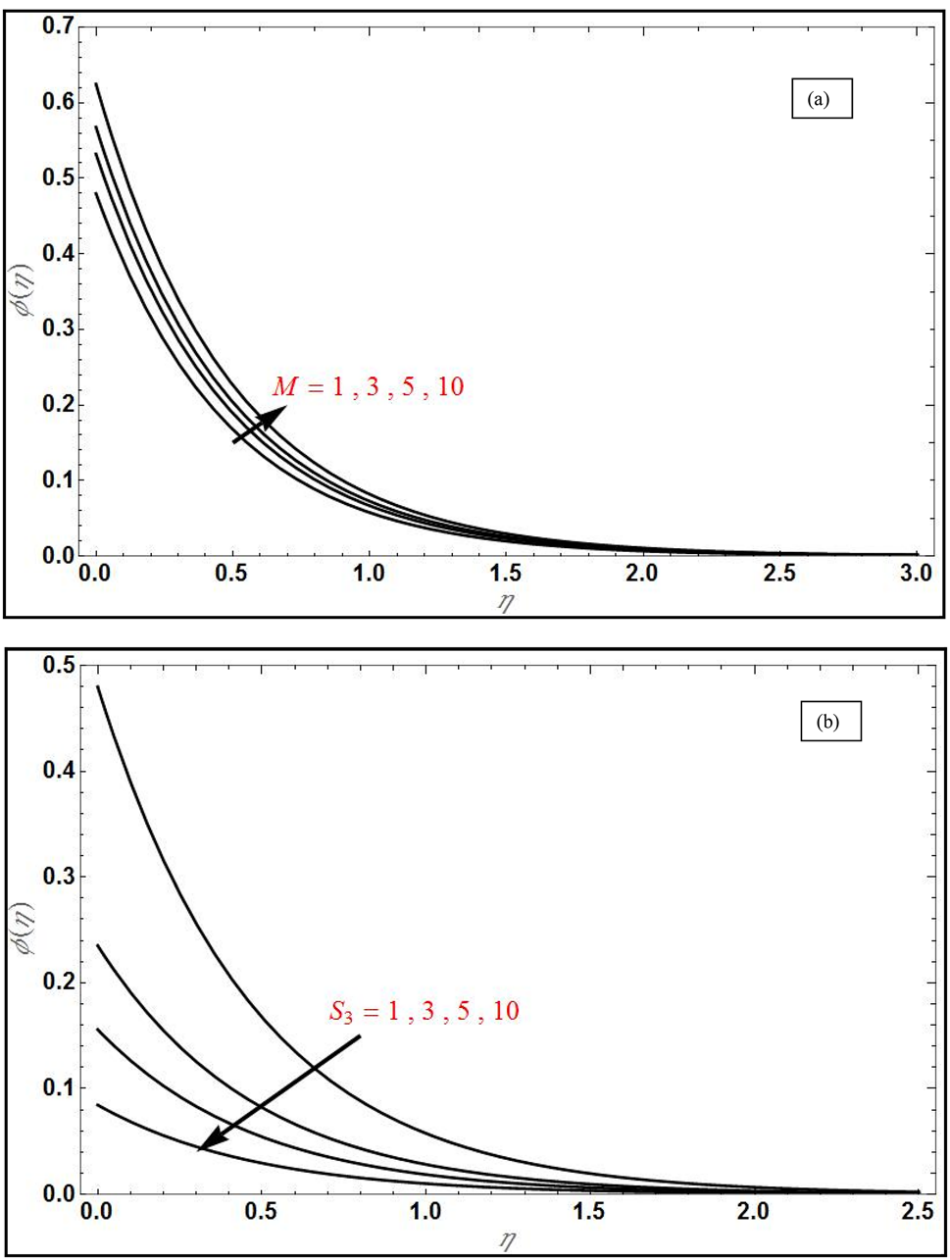
Concentration profile $\phi \left(\eta \right)$ for varying values of (**a**) magnetic parameter $\left( M \right)$ with $S_{3} = 1$ and in (**b**) for varying values of $S_{3}$ with $M = 1$ for suction case $\left( {V_{C} = 1} \right)$ with $Da^{ - 1} = \Lambda = 1$, $Sc = 2$, $S_{1} = 1,S_{2} = - 1\,$, $\varphi_{1} = 0.1,\varphi_{2} = 0.04$.

**Figure 6:**
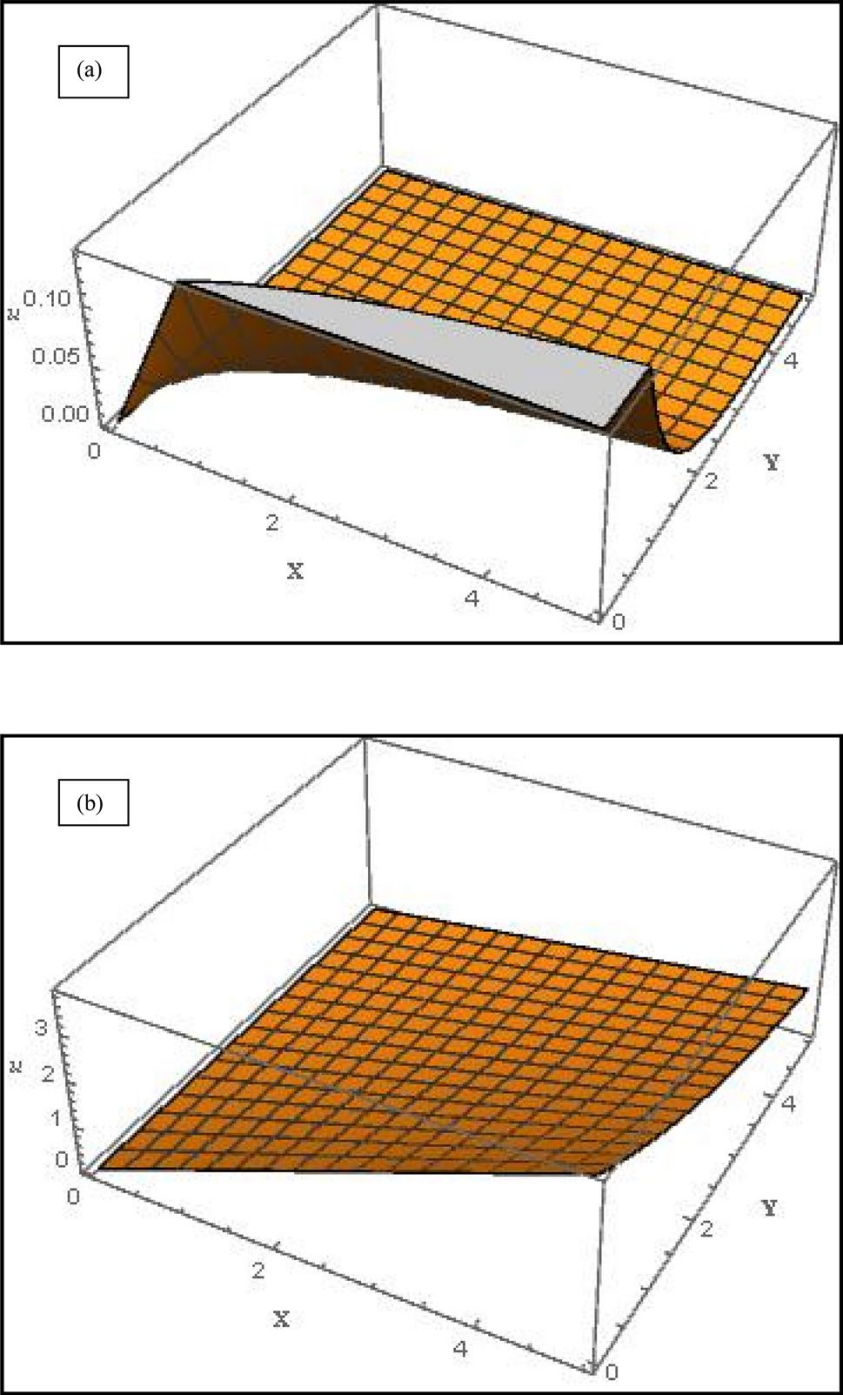
3-D graph of velocity profile along *x*-direction for (**a**) suction case $\left( {V_{C} = 1} \right)$ and (**b**) injection case $\left( {V_{C} = - 1} \right)$ with $M = Da^{ - 1} = \Lambda = 1$, $S_{1} = 1,S_{2} = - 1$, $\varphi_{1} = 0.1,\varphi_{2} = 0.04$.

**Figure 7:**
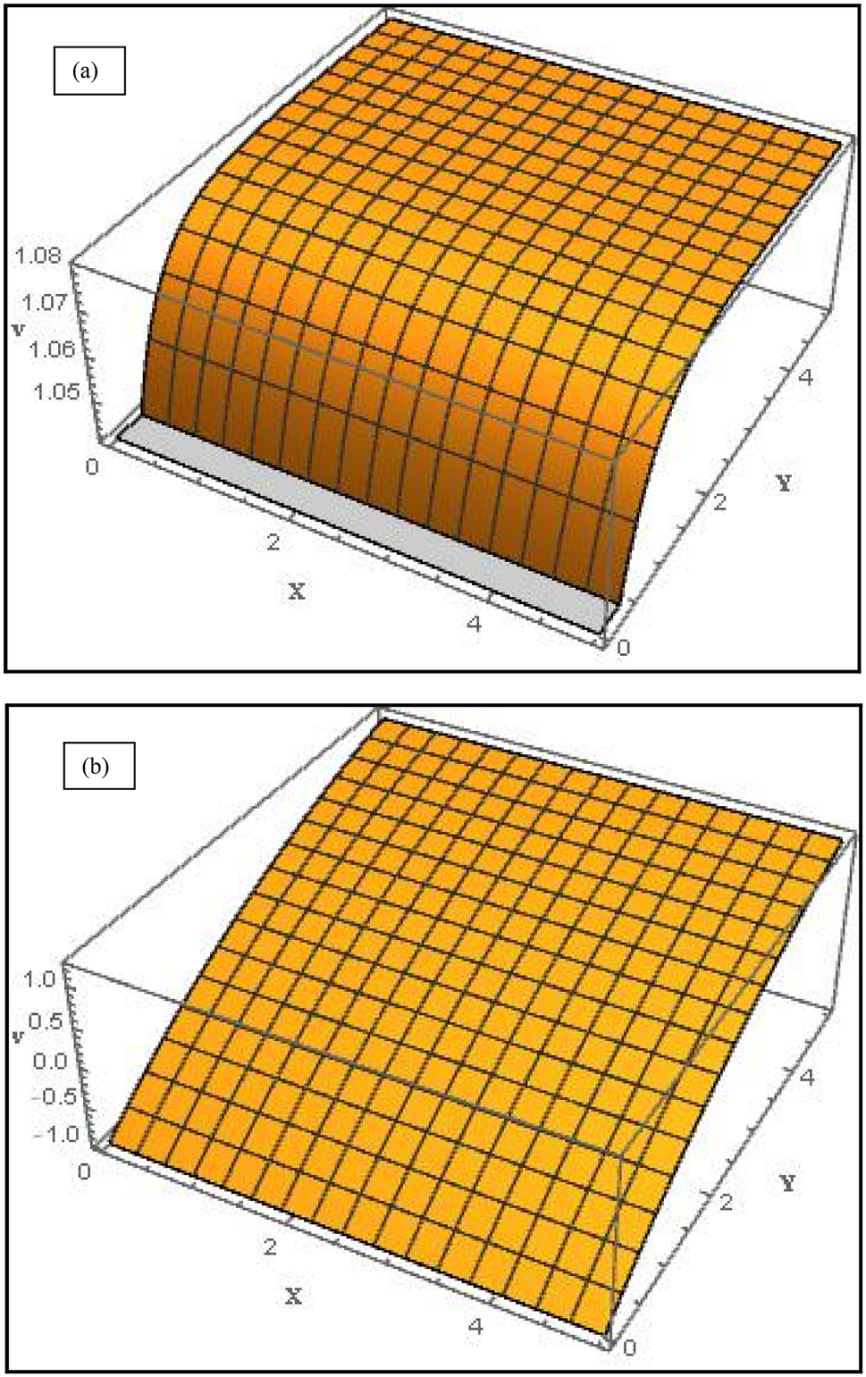
3-D graph of velocity profile along *y*-direction for (**a**) suction case $\left( {V_{C} = 1} \right)$ and (**b**) injection case $\left( {V_{C} = - 1} \right)$ with $M = Da^{ - 1} = \Lambda = 1$, $S_{1} = 1,S_{2} = - 1$, $\varphi_{1} = 0.1,\varphi_{2} = 0.04$.

The system of PDEs describing the HNF magnetic stretching sheet flow problem has been converted into a set of nonlinear ODEs with constant coefficients by taking the suitable similarity transformations for velocity and concentration. The analytical solution for velocity has been obtained in exponential form and that for the concentration field has been derived in terms of incomplete gamma function by the usage of LT. Using appropriate symbolic software e.g. MATLAB, the closed-form solutions can then be evaluated for different parameter values and plotted against transverse coordinate. The numerical results based on this process are visualized in Figs. 2, 3, 4, 5, 6 and 7 for the influence of constant magnetic field, mass transpiration, Navier’s slip and solutal slip effects. Copper-Alumina HNFconsidered.

Figure 2 portrays the deviation of transverse velocity with various *M* for suction case in (a) and injection case in (b) and reveals that, the transverse velocity will reduce with the increase in *M* for both cases. The same effect is evident in Fig. 3 for axial velocity, where these graphs reveals that the velocity boundary layer will increase in thickness with an increase in magnetic field due to the inhibiting effect of the Lorentz magnetic drag force which acts orthogonally to the applied magnetic field. This provokes a strong damping impact on the boundary layer flow. Initially transverse velocity will increase rapidly and becomes constant after some value of $\eta$ for both the cases, while the axial velocity initially decreases exponentially and becomes zero after some value of $\eta$. Axial velocity is always positive for both suction and injection cases (Fig. 3); however transverse velocity is only positive in the case of suction ($V_{C} = 1$) and evidently at large magnetic interaction parameter values velocity assumes negative values since blowing combined with strong Lorentz force (the effect is most prominent at high *M* values) induces flow reversal (back flow) in the boundary layer (Fig. 2b).

Figure 4a illustrates the skin friction distribution versus wall suction velocity $\left( {V_{C} > 0} \right)$ for various values of $Da^{ - 1}$. The skin friction decreases strongly with increment in suction effect. Similarly, there is a considerable drop produced in skin friction with greater values of $Da^{ - 1}$. This parameter which is the inverse Darcy number features in the linear Darcian impedance term, $-\left({\delta }_{2}D{a}^{-1}\right){f}_{\eta }$ in the transformed momentum Eq. (10). As $Da^{ - 1}$ increases, i.e. the medium possesses lower permeability so that there are more solid fibers of the porous matrix. The Darcian drag force is therefore elevated and this generates a concomitant deceleration in the transverse flow manifesting in depletion in the skin friction at the coating surface. The thickness of momentum boundary layer is therefore increased with increment in inverse Darcy number, $Da^{ - 1}$*.* However despite the marked retardation induced in the transverse flow, backflow is never caused i.e. there is no change in polarity of the skin friction. The suction effect clearly causes significant adherence of the momentum boundary layer in the NF flow to the sheet (wall) surface. Again however it does not produce flow reversal. The case of $V_{C} = 0$ corresponds to a solid wall i.e. absence of perforations and clearly results in the maximum skin friction computed. Effectively therefore strong wall suction and lower permeability of the porous medium successfully damp the boundary layer flow and this is advantageous in flow control operations in NF materials processing as noted by Shukla et al*.*^36^, among others. In all cases, skin friction gradually decays in the free stream and asymptotically smooth profiles are computed which validate that the sufficiently large infinity B.C has been imposed in the computations. Figure 4b portrays the evolution in skin friction as the function of magnetic parameter, *M* for various values of $V_{C}$. A very sharp decrement is observed initially for low values of M and thereafter profiles decay smoothly to the free stream. Significant suppression in skin friction is produced with more magnetic field effect verifying the earlier results presented for damping with enhanced Lorentz hydromagnetic body force, which is simulated with the term, $-\left({\delta }_{3}M\right){f}_{\eta }$ in Eq. (10).Again, a strong deceleration in the flow (i.e. depletion in skin friction) is induced with more suction effect $\left( {V_{C} } \right)$. The thickness of momentum boundary layer is therefore decreased with both elevation in magnetic field and suction, and again both these effects are excellent mechanisms for flow regulation in coating boundary layers.

Figure 5a displays the evolution in concentration field for various *M* values in the case of suction at the wall $\left( {V_{C} = 1} \right)$. Although MHD effects do not feature directly in the concentration Eq. (11), the term $+Scf{\varphi }_{\eta }$ couples the nanoparticle concentration ${, \varphi }_{\eta }$ to the momentum Eq. (10). Magnetic Lorentz force therefore indirectly influences the nanoparticle species diffusion field which is enhanced with stronger *M* values. Therefore the concentration boundary layer thickness is also enhanced with increment in *M*. In all cases the concentration decays exponentially from the highest value at the wall $\left( {\eta = 0} \right)$ and converges to zero in the free stream. Figure 5b the effect of solutal slip $S_{3}$ on concentration field; evidently there is a strong decrease in concentration magnitudes with greater solutal slip.$S_{3}$ features only in the wall concentration B.C, $\phi \left(0\right)=1+{S}_{3}{\phi }_{\eta }\left(0\right)$. With greater slip there is a delay in mass transfer from the wall to the NF. This results in reduction also in a thinner concentration boundary layer. A same effect has been observed by Shukla et al*.*^42^. The implication is that when solutal slip is either low or ignored, the concentration values are over-predicted as is the concentration boundary layer thickness. Concentration converges to zero more quickly than in previous plot. The 3-D graphs for velocities along *x*- (axial) and *y*-(transverse) directions are shown in Figs. 6 and 7 for both the suction and injection cases. At low values of *y*, *x*-direction velocities are maximized whereas they are minimized at low values of *x* (Fig. 6a, *suction case*). In the injection case (Fig. 6b), velocity is maximum at only high values of *x* and low value of *y*. Figure 7a shows that with suction, *y*-direction velocity is maximized at large *y* and minimized at lower values for all *x* values*.* However, with injection Fig. 7b shows a similar topology to Fig. 7a, although the profile is more linear rather than the strong parabolic profile in Fig. 7a. The effects of suction and injection are therefore prominently demonstrated in these 3-D profiles.

## Concluding remarks

The mathematical model for hybrid nanofluid boundary layer slip flow and mass transfer from a stretching surface in porous media with the impact of magnetic field and chemical reaction has been developed. Darcy’s law is deployed. Momentum slips of both first and second order are included as is solutal slip. Using appropriate transformations, analytical solutions have been derived in terms of exponential and gamma functions for the velocity and concentration field, under proper boundary conditions by the application of the Laplace transformation technique. A parametric study of selected parameters i.e. magnetic body force parameter, inverse Darcy number, wall transpiration (suction and injection) and wall solutal slip on transverse and axial velocity, skin friction and concentration distributions is visualized graphically. Magnetic field suppresses velocity and increases the thickness of the hydrodynamic boundary layer. The flow is accelerated with reduction in inverse Darcy number and stronger suction direct to reduce in skin friction. The analysis provides a good foundation for further investigations using numerical methods. The main findings of observed results can be summarized as below:

- The transverse velocity is always positive while the axial velocity may be negative also depending on the strength of the magnetic field.
- With increasing inverse Darcy number (i.e. decreasing permeability) the velocity is reduced, and momentum boundary layer thickness is increased.
- The skin friction is generally suppressed with increasing suction and increased with injection.
- The concentration magnitudes are boosted with magnetic field whereas they are depleted with increasing solutal slip.

The present nanofluid hydromagnetic coating model has shown that the Laplace transform method has many useful applications in nanofluid transport modeling. However, this study has considered only *Newtonian* hybrid nanofluid flow. Future studies may implement *micropolar non-Newtonian models* and will be communicated imminently.

## Latin symbols

*a*: Constant
*A, B*: Constants
*B*_0_: Applied magnetic field (w m^−^^2^)
*C*: Nanoparticles concentration field (mol m^−^^3^)
*C*_*w*_: Wall concentration (mol m^−^^3^)
*C*_∞_: Concentration away from the wall (mol m^−^^3^)
*D*_*B*_: Solutal diffusivity (m^2^ s^−^^1^)
*Da*^−1^: Inverse Darcy number $\left( { = \frac{{\nu _{f} }}{{\kappa a}}} \right)$
*f*: Similarity variable
*k*_*c*_: Rate constant of chemical reaction
*K*: Knudsen number
*K*_1_, *L*_1_, *L*_2_: Slip parameter functions
*M*: Magnetic interaction parameter $\left( { = \frac{{\sigma _{f} B_{0}^{2} }}{{\rho _{f} a}}} \right)$
*S*_1_ ≥ 0: First order slip
*S*_2_ ≤ 0: Second order slip
*S*_3_: Solutal slip parameter
*Sc*: Schmidt number $\left( { = \frac{{\nu _{f} }}{{D_{B} }}} \right)$
*u*, v: Velocity along *x*- and *y*-direction (m s^−^^1^)
*V*_C_: Mass transpiration parameter
v_*w*_: Wall mass transfer velocity (m s^−^^1^)
*x*, *y*: Co-ordinate axes (m)

## Greek symbols

*α*: Molecular free path
*β*: Chemical reaction parameter $\left( { = \frac{{k_{c} }}{a}} \right)$
*γ*: Coefficient of momentum accommodation
*δ*_1_: Density ratio $$\left( { = \frac{{\rho _{{hnf}} }}{{\rho _{f} }}} \right)$$
*δ*_2_: Dynamic viscosity ratio $\left( { = \frac{{\mu _{{hnf}} }}{{\mu _{f} }}} \right)$
*δ*_3_: Electrical conductivity ratio $\left( { = \frac{{\sigma _{{hnf}} }}{{\sigma _{f} }}} \right)$
*η*: Similarity variable
*κ*: Permeability of porous medium
*λ*: Exponent
*μ*: Dynamic viscosity (kg m^−^^1^ s^−^^1^)
*ν*: Kinematic viscosity (m^2^ s^−^^1^)
*ν*_*eff*_: Effective kinematic viscosity $\left( { = \frac{{\mu _{{eff}} }}{{\rho _{f} }}} \right)$
*ξ*: Change of variable
*ρ*: Density (kg m^−3^)
σ: Electrical conductivity (S m^−1^)
*ϕ*: Similarity variable for concentration
*ψ*: Stream function
Γ: Incomplete gamma function
Λ: Brinkman viscosity ratio $\left( { = \frac{{\mu _{{eff}} }}{{\mu _{f} }}} \right)$

## Subscripts

*f*: Parameter of base fluid
*hnf*: Parameter of hybrid nanofluid

## Abbreviations

BC: Boundary conditions
CNTs: Carbon nanotubes
HNF: Hybrid nanofluid
L.T: Laplace transformation
MHD: Magnetohydrodynamics
NF: Nanofluid
ODEs: Ordinary differential equation
PDEs: Partial differential equations
